# Demonstration of Microwave Resonators and Double Quantum Dots on Optimized Reverse-Graded Ge/SiGe Heterostructures

**DOI:** 10.1021/acsaelm.4c00654

**Published:** 2024-06-26

**Authors:** Arianna Nigro, Eric Jutzi, Fabian Oppliger, Franco De Palma, Christian Olsen, Alicia Ruiz-Caridad, Gerard Gadea, Pasquale Scarlino, Ilaria Zardo, Andrea Hofmann

**Affiliations:** † Physics Department, University of Basel, Klingelbergstrasse 82, Basel CH-4056, Switzerland; ‡ Hybrid Quantum Circuits Laboratory, Institute of Physics, École Polytechnique Fédérale de Lausanne (EPFL), Lausanne 1015, Switzerland; § Swiss Nanoscience Institute, Klingelbergstrasse 82, Basel CH-4056, Switzerland

**Keywords:** germanium quantum well, microwave resonator, quantum dot, transport experiments, semiconductor qubit

## Abstract

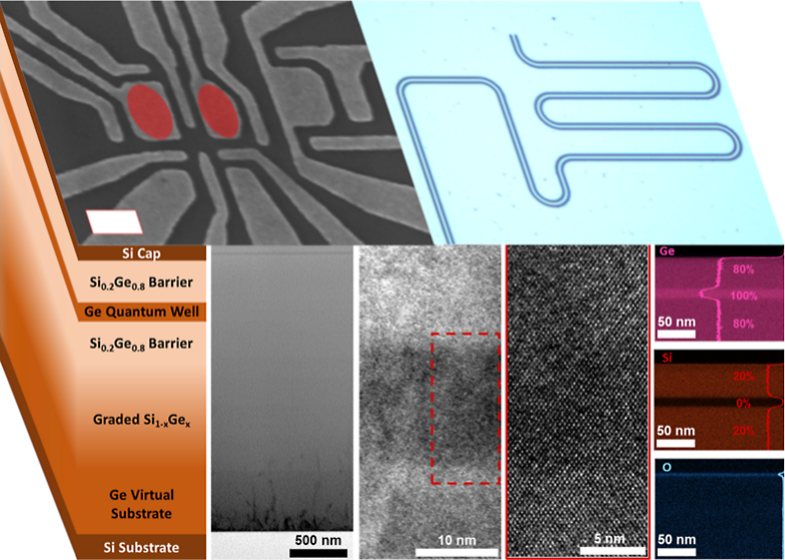

One of the most promising platforms for the realization of spin-based quantum computing are planar germanium quantum wells embedded between silicon–germanium barriers. To achieve comparably thin stacks with little surface roughness, this type of heterostructure can be grown using the so-called reverse linear grading approach, where the growth starts with a virtual germanium substrate followed by a graded silicon–germanium alloy with an increasing silicon content. However, the compatibility of such reverse-graded heterostructures with superconducting microwave resonators has not yet been demonstrated. Here, we report on the successful realization of well-controlled double quantum dots and high-quality coplanar waveguide resonators on the same reverse-graded Ge/SiGe heterostructure.

## Introduction

Strained germanium (Ge) quantum wells (QWs) embedded in planar silicon–germanium (SiGe) heterostructures have been proven to be excellent candidates for a wide range of applications,^1−4^ among which quantum computing^5^ is one of the most prominent. High mobilities^6^ and light effective masses^7^ of the holes, together with a strong and gate-tunable spin–orbit interaction,^8^ have been exploited to fabricate devices showing low disorder,^9^ high gate fidelities,^10^ and fast qubit operations.^11^ These intrinsic properties, combined with a high scalability potential and compatibility with conventional CMOS fabrication processes, allow the realization of arrays of qubits.^12,13^ The performance of these devices critically depends on the crystalline quality of the Ge QW and Ge-rich barrier layers, which is complicated by a large lattice mismatch with the CMOS-compatible Si substrate. Achieving high Ge contents by forward grading, i.e., slowly increasing the Ge content, requires several micrometers of material leading to elevated roughness. In contrast, much less material is used and better morphology is achieved using reverse linear grading, where a Ge layer is grown on top of a Si substrate, which is subsequently employed as a virtual substrate for the deposition of Ge-rich SiGe layers.^14^ Improvements in the surface morphology and in the residual density of dislocations translated into record values for the hole mobility above 1 × 10^6^ cm^2^/(V s).^6,15,16^

Recently, high levels of control of spin qubits in planar Ge/SiGe heterostructures have been demonstrated using both Loss–DiVincenzo^13,17^ and singlet–triplet^18,19^ encoding. It is interesting to note that a majority of quantum dot (QD) qubit works have been published on reverse-graded heterostructures.^11−13,18,20^ Meanwhile, important results have also been obtained on forward-graded substrates,^19,21^ though the last hole regime has not been routinely achieved. Scaling up the number of spin qubits in gate-defined semiconducting QDs, however, presents many challenges. For example, the electron–electron interaction acts over a very short range (∼100 nm), forcing compact dot array designs, thus complicating manipulation and readout schemes. Circuit quantum electrodynamics (cQED), describing the physics of a two-level system (TLS) coupled to single microwave photons, represents one possible solution to overcome the short-range interaction and holds enormous potential for various applications in quantum technology.^22^ These applications include long-range interactions between distant QD qubits,^23−25^ rapid and high-fidelity charge and spin state detection,^26,27^ analogue quantum simulations of open quantum systems,^28^ and the development of gigahertz photodetectors.^29^ However, performing cQED experiments sets high demands not only on the fabrication of the devices but also on the heterostructure material itself. In particular, the integration of microwave superconducting resonators has been demonstrated on forward-graded^21^ but not on reverse-graded Ge/SiGe heterostructures, which have so far been the material of choice for achieving QDs with a control down to the last holes^10,30^ and especially for building many-qubit devices.^11−13^

In this work, we present the successful realization of fully controllable double quantum dots (DQDs) and 50 Ω coplanar resonators on highly crystalline Ge/SiGe planar heterostructures grown by using the reverse linear grading approach on a Si substrate. As a proof of concept demonstration for hybrid devices on such substrates, we present superconducting resonators with internal quality factors *Q*_int_ in the order of 1000. Meanwhile, the quality of the DQDs formed on the same heterostructure material is demonstrated by a regular, uninterrupted charge stability diagram, as well as the control over single charging events down to the last holes in both QDs.

## Results and Discussion

### QW Heterostructure Growth

**Figure 1:**
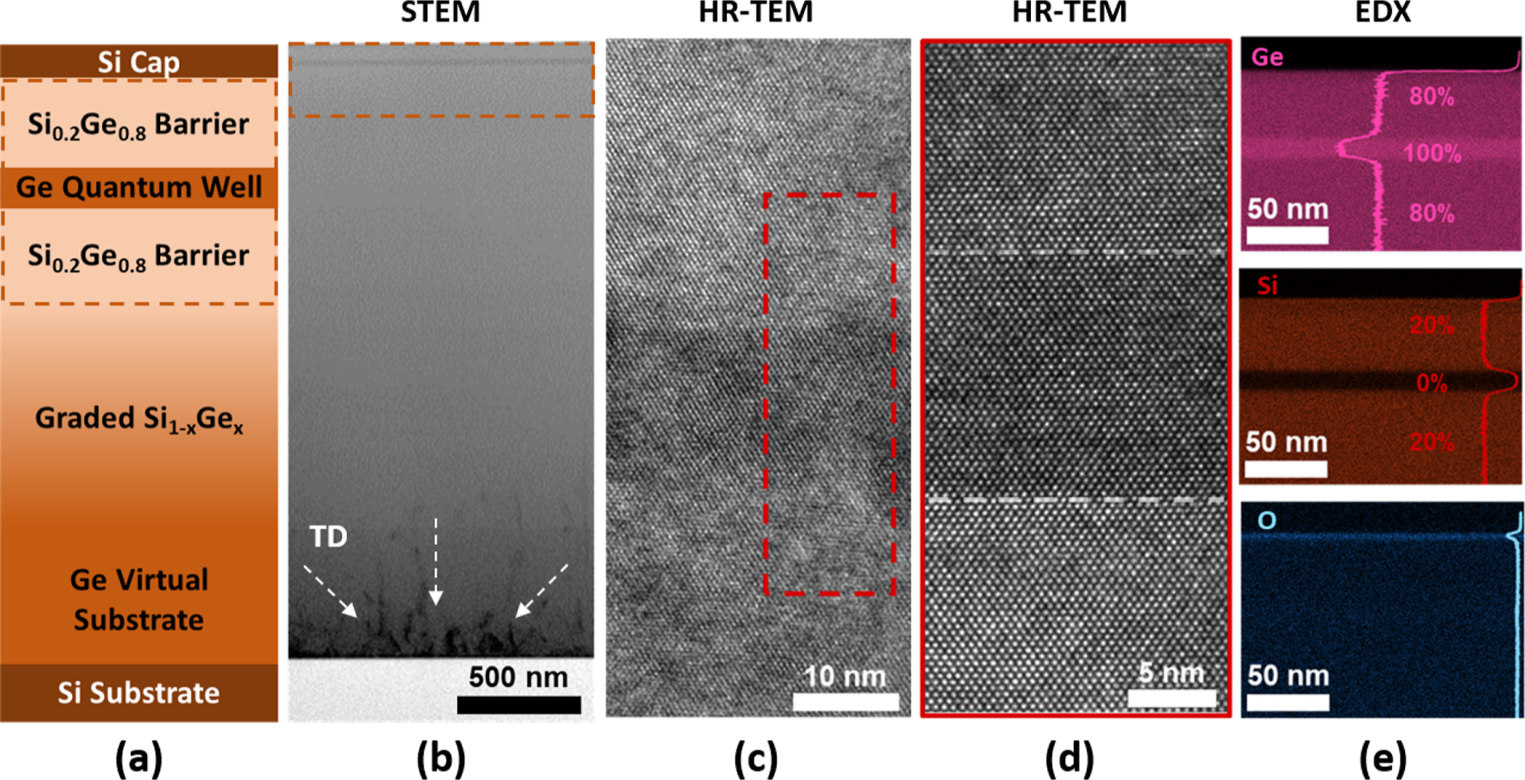
(a) Schematic representation of the Ge/SiGe heterostructure. Please note that this panel is not in scale. (b) Scanning TEM of the Ge/SiGe heterostructure. The white arrows highlight threading dislocations. (c) HR-TEM of the QW region. (d) HR-TEM of the region highlighted in (c). (e) EDX maps of the Si_0.2_Ge_0.8_ barriers and Ge QW highlighting the content of Si, Ge, and O (from top to bottom) in the different layers.

In order to assess the main morphological and crystalline properties of the heterostructure, schematically depicted in Figure 1, a cross section was analyzed through scanning electron tunnelling microscopy (TEM) at different magnifications (Figure 1). Figure 1b displays how the threading dislocations are successfully confined in the lowest layers, less than 1 μm of the heterostructure, as a result of the reverse linear grading growth approach. In particular, the threading dislocation density (TDD) is higher in the first tens of nanometers of the Ge virtual substrate, and it decreases when increasing the thickness of the Ge virtual substrate itself, most likely as a consequence of annihilation processes through interaction.^31,32^ A quantification of the TDD evaluated by etch pit count (EPC) reveals a density of (6.0 ± 0.8) × 10^8^ cm^–2^ on top of the graded SiGe layer. The quality of the interfaces between the Ge QW and the two Si_0.2_Ge_0.8_ barriers was verified through high-resolution (HR)-TEM, as shown in Figure 1c,d. In particular, both materials are observed to be single crystalline, and no defect can be identified in the region of interest. Finally, the chemical composition of the layers forming the QW stack is displayed in Figure 1e. The energy dispersive X-ray (EDX) maps corresponding to the signals of Ge and Si show the achievement of a uniform chemical composition for the Si_0.2_Ge_0.8_ alloy and sharp transitions between the Ge QW and Si_0.2_Ge_0.8_ barriers. To quantify these interfaces, the concentration profiles of Ge and Si displayed in Figure 1e were fitted with an error function.^9,33^ The analysis was performed on a set of five samples, providing an upper bound value of (1.2 ± 0.3) nm for the interfacial abruptness between the bottom Si_0.2_Ge_0.8_ barrier and the Ge QW and of (1.6 ± 0.2) nm between the Ge QW and the top Si_0.2_Ge_0.8_ barrier. Both values fall within the range of results previously reported in the literature.^9,34^ Finally, the EDX map for the O signal confirms the post-growth oxidation of the Si capping layer following the exposure of the material to air.

### Quantum Dots

**Figure 2:**
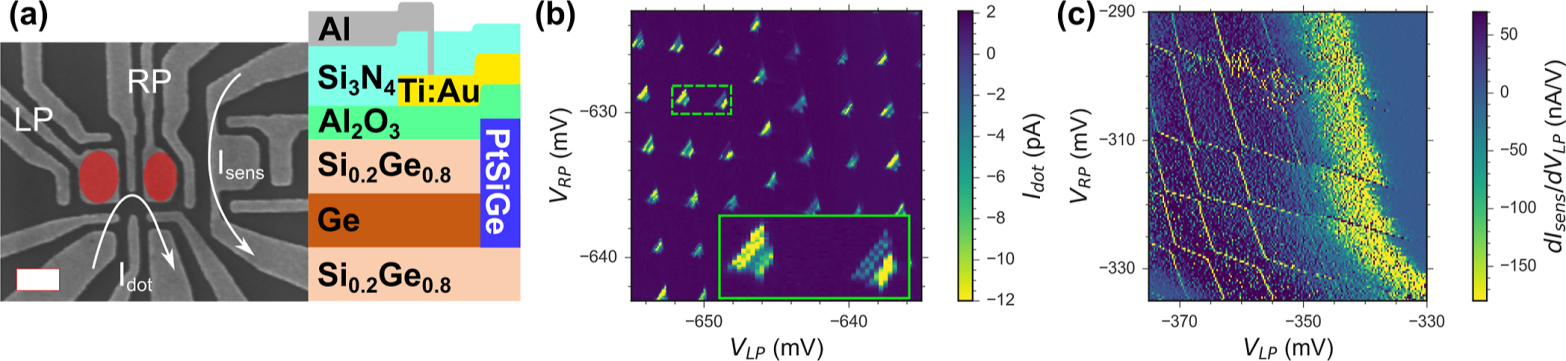
(a) SEM image of a device nominally identical to the two used for this study, indicating *I*_dot_ as the current through the DQD and *I*_sens_ as the current through the SHT. The scale bar corresponds to 200 nm. Two QDs are defined by the left plunger (LP) and right plunger (RP) gates. The schematic cross section shows the fabrication steps performed. (b) Measurement of direct current *I*_dot_ through the DQD showing a regular pattern of bias triangles. The inset on the bottom right shows a close-up of the dashed region. (c) Transconductance d*I*_sens_/d*V*_LP_ of the SHT charge sensor showing the last charge transitions on both QDs.

The electronic transport properties of the heterostructure are characterized in detail in a previous study.^35^ In accordance with the designed undoped heterostructure, transport was only observed after inducing a 2D hole gas using negative gate voltages, and a maximal Hall mobility of μ = 6.4 × 10^4^ cm^2^/(V s) was extracted at a density of *n* = 2.46 × 10^10^ cm^–2^.^35^ However, for QD qubit applications, the maximal mobility is not necessarily a good figure of merit, as it does not allow conclusions about the low-density regime in which the QDs are operated. More importantly, a homogeneous low-energy-potential landscape is required. The percolation density *n*_p_ marks the onset of metallic conduction. With the extracted *n*_p_ = 2.3 × 10^10^ cm^–2^ in the employed heterostructure,^35^ we have control over the density down to a single hole per (66 nm)^2^. This suggests, on the one hand, that we expect full control of QDs down to the last hole regime and, on the other hand, that the density of deep impurities is low. In order to confirm these expectations, we fabricate simple single-layer DQD devices with a single hole transistor (SHT), as shown in Figure 2a. The gate-layer has been optimized to allow for emptying the QDs down to the last holes. Transport through the DQD, *I*_dot_, is shown in Figure 2b, while the transconductance d*I*_sens_/d*V*_LP_ through the SHT is shown in Figure 2c. In the direct transport, a regular pattern of bias triangles is visible over large gate voltage regions without observing any signatures of spurious dots. The SHT, used as a charge sensor, shows the charge stability diagram of the DQD, demonstrating our control of the DQD down to the last hole regime. This and similar data measured reproducibly on various other devices highlight the high quality of the heterostructure.

### Resonators

**Figure 3:**
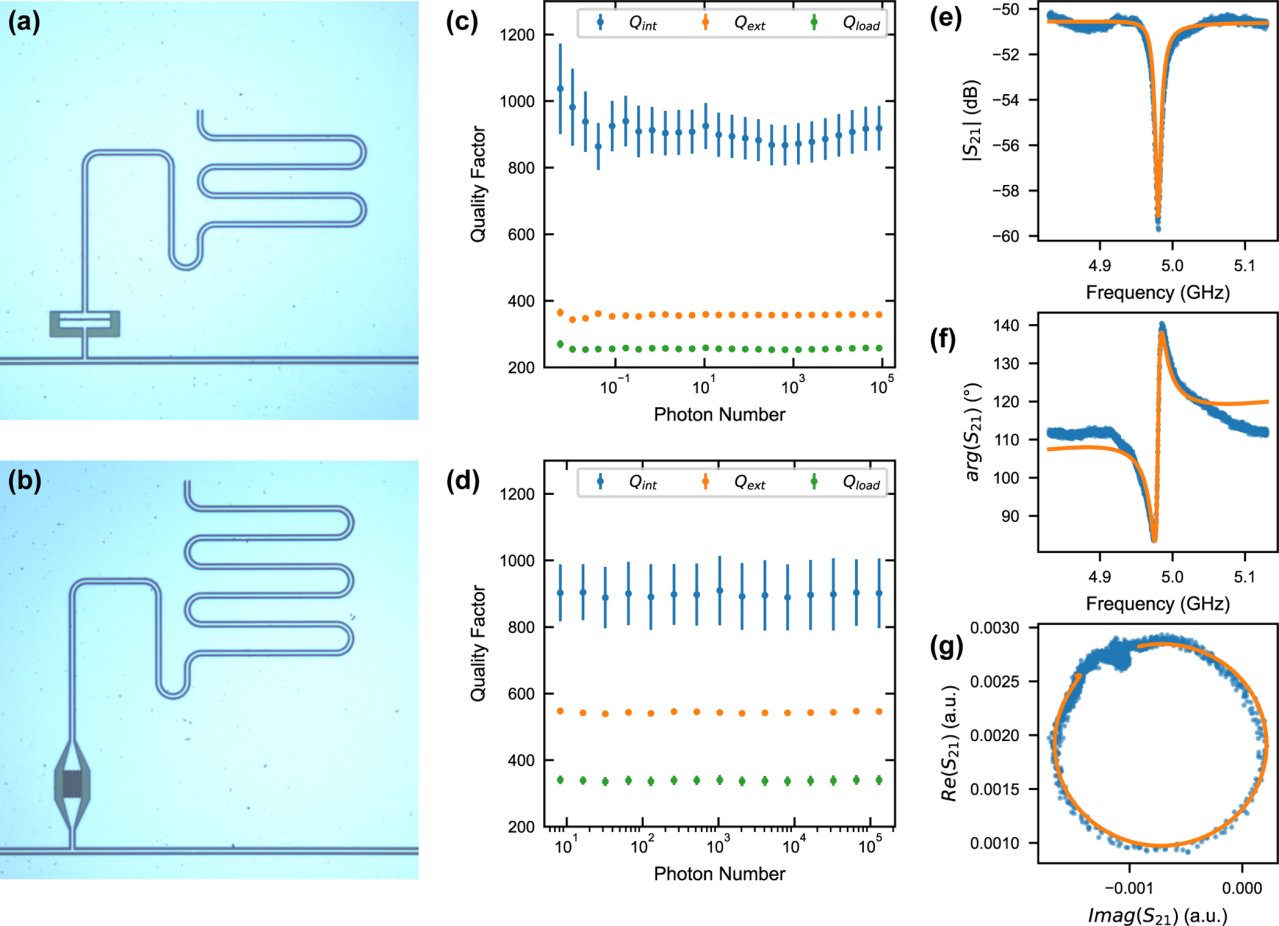
(a,b) Optical micrographs of the CPW resonators with two different coupling capacitor shapes, coplanar plates (a) and interdigitated fingers (b). (c,d) Internal, external, and loaded quality factors as a function of average photon number for the resonators shown in panels (a,b), respectively. (e) Magnitude, (f) phase, and (g) complex values of the feedline transmission of a selected resonance with measured values in blue and a numerical fit in orange.

In order to estimate the compatibility of the Ge/SiGe heterostructure with microwave resonators, 50 Ω coplanar waveguide (CPW) resonators were fabricated. They were designed in a notch-type configuration, by coupling them to a 50 Ω transmission line via a coupling capacitance *C*_ext_, to allow multiplexing and to accurately extract the internal quality factor *Q*_int_, a measure for the intrinsic loss rate of the resonators.^36^ The devices contain two 50 Ω transmission lines with three resonators (Figure 3a,b). We chose the length of the three resonators to have their resonance frequencies, respectively, around 5, 6, and 7 GHz.

Two different types of coupling capacitors were employed: coplanar plates and interdigitated fingers, as shown in Figure 3a,b, respectively. Such different *C*_ext_ designs allow us to reach coupling rates κ_ext_ ranging from ∼5–10 MHz, for the six different realizations, ensuring that the resonators are adequately coupled and exhibit discernible responses. The measured feedline transmission (*S*_21_) was fitted to a notch-type input–output model using the resonator_tools Python package,^37^ as shown in Figure 3e–g. Internal quality factors of *Q*_int_ ∼ 800–1000 were extracted for most devices, with a few outliers attributed to coupling to standing waves. This results in a resonator line width of <10 MHz, comparable to what was obtained in initial cavity-QD hybrid devices on GaAs^38^ and silicon nanowire metal–oxide semiconductor (MOS),^39^ potentially enabling first proof-of-concept hybrid cQED experiments with charge and spin qubits defined in planar-Ge.

Common sources of losses for superconducting microwave resonators fabricated on semiconducting heterostructures are represented by capacitive coupling to residual conductive regions within the substrate or to an ensemble of TLS in the dielectric or nearby interfaces, as well as resistive loss due to quasiparticle excitations.^36,40^ TLS losses are usually characterized by a specific power dependence of *Q*_int_, with an increasing *Q*_int_ for higher intracavity power.^36^ Quasiparticles can be excited by infrared irradiation from hotter stages of the cryogenic setup due to insufficient shielding^41^ or by phonon-mediated heating induced by a high-power readout signal.^42^

In our case, power-dependent measurements shown in Figure 3c,d reveal that *Q*_int_ does not significantly depend on the average photon number in the resonator. Furthermore, measurements of the same resonators realized on an intrinsic Si substrate and measured with the same microwave packaging exhibited *Q*_int_ > 10^4^ with the lower bound originating from significant overcoupling to the feedline as κ_ext_ has been optimized for the Ge/SiGe heterostructure. Also, these resonators exhibited the typical TLS-induced power dependence of *Q*_int_. We can thus exclude TLS or infrared radiation-mediated heating as the main sources of losses and conclude that the primary loss mechanism stems from residual conductive losses from carriers that do not freeze out even at cryogenic temperatures or microwave-active defects within the heterostructure.

We then turn to investigate the probable origins of such conductive losses. To exclude free carriers in the QW, identical resonators were characterized on a substrate where the QW had been etched away by dry etching. No significant difference in *Q*_int_ was observed, confirming that no free carriers are present in the QW at cryogenic temperatures, consistent with the transport measurements reported above. Moreover, identical resonators were fabricated and characterized on a different heterostructure with a thicker Ge virtual substrate of ∼1.1 instead of ∼0.5 μm. The comparable value of *Q*_int_ ∼ 1000 and its similar behaviour with microwave power suggest that the majority of losses do not occur in the bulk of the virtual substrate either. This leaves the interface between the Si substrate and the Ge virtual substrate with its large number of defects originating from the lattice mismatch between Si and Ge as the most likely loss source.

## Conclusions

We demonstrate the compatibility of crystalline Ge/SiGe heterostructures grown on top of a reverse-graded virtual substrate with DQDs and CPW resonators. In particular, the quantum well is crystalline and is limited by sharp interfaces to the barriers, the DQDs are controlled down to the last holes using a simple single-layer layout, and resonators with quality factors ∼1000 are obtained on heterostructures on top of 0.5 to 1 μm thick Ge virtual substrates. We find that the bottom Ge–Si interface is most likely the dominant loss source, highlighting the importance of well-controlled growth of the virtual substrate. These results demonstrate the feasibility of combining QD qubits with resonators on reverse-graded Ge/SiGe heterostructures. Additional studies are needed to further improve the quality of superconducting resonators on reverse-graded GeSi heterostructures. We note that a recent preprint^43^ reports the coupling of QDs to a superconducting microwave resonator, though no information about the heterostructure growth is given.

## Experimental Section

### Material Growth and Structural Characterization

The Ge/SiGe heterostructures were epitaxially grown by a cold wall chemical vapor deposition (CVD) using a PlasmaPro 100 Nanofab reactor equipped with a showerhead (Oxford Instruments, base pressure <0.5 mTorr), commercial germane (GeH_4_, Pangas, 99.999%) and silane (SiH_4_, Pangas, 99.999%) as gaseous precursors, and hydrogen (H_2_, Pangas, 99.999%) as diluting gas. Si(100) wafers (2 in., floating zone, undoped, resistivity >10,000 Ω cm) were chosen as substrates for the growth. Before being loaded into the reactor, they were cleaned through a 1 min dip in 2% HF aqueous solution to remove the native oxide and finally rinsed in deionized water and isopropanol. The heterostructures were grown following the reverse linear grading approach, as shown schematically in Figure 1a. The Ge virtual substrate, which constitutes the first layer of the stack, was grown in two steps: a thin Ge seed layer (∼100 nm) was deposited at 400 °C and 30 mTorr GeH_4_ partial pressure by employing a dilution of 0.1% GeH_4_ in H_2_. It was complemented by a thicker (∼400 nm) Ge layer deposited at 500 °C and 400 mTorr GeH_4_ partial pressure by employing a dilution of 5% GeH_4_ in H_2_. A reverse linearly graded Si_1–*x*_Ge_*x*_ alloy (∼750 nm), in which the Ge content was linearly decreased from 100 to 80%, was grown on top of the Ge virtual substrate at 500 °C and a GeH_4_ partial pressure in a range from 400 to 450 mTorr with a dilution of 5% GeH_4_ in H_2_. In particular, the GeH_4_ and H_2_ flow rates were kept constant, and the SiH_4_ flow rate was increased while growing, leading to a grading rate of 10% per μm. The same deposition conditions as for the final nanometers of the graded alloy were employed for the bottom and top Si_0.2_Ge_0.8_ barriers (∼300 and ∼55 nm, respectively). Between the Si_0.2_Ge_0.8_ barriers, the Ge QW (∼15 nm) was grown at 500 °C and 30 mTorr GeH_4_ partial pressure by employing a dilution of 1% GeH_4_ in H_2_. Finally, a protective Si capping layer (∼1.5 nm) was grown at 500 °C and 10 mTorr SiH_4_ partial pressure. The growth temperature of the topmost films was set to 500 °C in order to suppress intermixing between the SiGe barriers and the Ge QW.^44,45^ Moreover, a 15s-long H_2_ purging step (500 °C, 100 mTorr), interposed between the growth of the four final layers, allowed us to enhance the sharpness at the interfaces.^46^

The morphology and crystalline quality of the materials, as well as the chemical composition of the different layers, were investigated using a Jeol JEM-F200 cFEG TEM operating at 200 kV by performing HR-TEM, scanning TEM, and EDX analysis. Prior to imaging, electron transparent cross-sectional lamellas of the heterostructures were fabricated by means of focused ion beam using a FEI Helios Nano Lab 650 microscope operated at 30 and 5 kV voltages. A selective chemical etching process, namely, EPC, was used to reveal threading dislocations and to quantify the TDD. The etching solution employed was obtained by diluting 60 mg of I_2_ in 52 mL of a mixture of HF 2.3%:HNO_3_ 50%:CH_3_COOH 100% in a volume ratio 10:2:2. The samples were etched for 2 min at room temperature and analyzed by scanning electron microscopy (SEM).

### Fabrication

#### Quantum Dots

The fabrication is similar to that of hallbars described in our previous work.^35^ In the first step, ohmic contacts were deposited using electron-beam evaporation. Before evaporating the Pt contacts, the native oxide underneath was locally removed by in situ Ar milling. After lift-off of the contacts, the native silicon oxide was removed globally using a 60 s dip in 2.3% HF. The sample was rinsed in deionized water and covered in isopropanol during the transport to the atomic layer deposition chamber, where 30 nm of Al_2_O_3_ was grown at 225 °C. The gate layer was patterned by electron-beam lithography and subsequent evaporation of Ti and Au by electron-beam evaporation. To protect the heterostructure from damage during wire-bonding, 210 nm of Si_3_N_4_ was deposited using plasma-enhanced CVD at 300 °C. This step additionally serves as an annealing step to diffuse the Pt into the QW to form ohmic contacts. Holes were subsequently etched into the dielectric layer using reactive ion etching to connect the gates and the bond pads. A schematic cross section of a finished device is shown in Figure 2a.

#### Superconducting Resonators

The superconducting resonators and the ground plane were fabricated in a single step using electron-beam lithography, electron-beam evaporation, and lift-off. The sample was first rinsed in acetone and isopropanol and then coated with a double-layer electron-beam resist (MMA/PMMA). A 120 nm thick layer of Al was then evaporated, followed by an oxidation step in situ to ensure a high-quality oxide that prevents the Al from further oxidizing by exposure to air. The lift-off process ensures that the resonator and QD fabrication are compatible. In some devices, the QW is etched away by reactive ion etching prior to patterning the resonator using a plasma based on SF_6_, CHF_3_, and O_2_.

## Data Availability

The data that support the findings on this study are openly available in ZENODO at https://zenodo.org/doi/10.5281/zenodo.10990400, Reference No. 10990401.
